# Effect of Different Metal Ions on the Biological Properties of Cefadroxil

**DOI:** 10.3390/ph2030184

**Published:** 2009-12-15

**Authors:** Sayed H. Auda, Ilka Knütter, Beate Bretschneider, Matthias Brandsch, Yahya Mrestani, Cornelia Große, Reinhard H. H. Neubert

**Affiliations:** 1 Institute of Pharmacy, Martin-Luther-University Halle-Wittenberg, Germany; 2 Department of Pharmaceutics and Industrial Pharmacy, Faculty of pharmacy, Al-Azhar, University, Assuit branch, Assuit, Egypt; Email: sayed.auda@yahoo.com (S.H.A.); 3 Membrane Transport Group, Biozentrum, Martin-Luther-University Halle-Wittenberg, Germany; Email: matthias.brandsch@biozentrum.uni-halle.de (M.B.); 4 Institute of Applied Dermatopharmacy, Martin-Luther-University Halle-Wittenberg, Germany; 5 Institute of Biology/Microbiology, Martin-Luther-University Halle-Wittenberg, Germany

**Keywords:** cefadroxil, metal ions, Caco-2, transepithelial flux, antibacterial activity

## Abstract

The effect of different metal ions on the intestinal transport and the antibacterial activity of cefadroxil [(6*R*,7*R*)-7-{[(2*R*)-2-amino-2-(4-hydroxyphenyl)acetyl]amino}-3-methyl-8-oxo-5-thia-1-azabicyclo[4.2.0]oct-2-ene-2-carboxylic acid] was investigated. The [^14^C]Gly-Sar uptake via PEPT1 was inhibited by Zn^2+^ and Cu^2+^ treatment in a concentration-dependent manner (K_i_ values 107 ± 23 and 19 ± 5 µM, respectively). Kinetic analysis showed that the K_t_ of Gly-Sar uptake was increased 2-fold in the presence of zinc sulphate (150 µM) whereas the V_max_ value were not affected suggesting that zinc ions inhibited Gly-Sar uptake by PEPT1 in a competitively manner. Ni^2+^ exhibited moderate inhibitory effect, whereas Co^2+^, Mg^2+^, Al^3+^ ions showed no inhibitory effect on Gly-Sar uptake via PEPT1. Subsequently, we examined the effect of Zn^2+^ and Al^3+^ ions on the transepithelial transport of cefadroxil across Caco-2 cells cultured on permeable supports. The results showed that zinc ions inhibited the transepithelial flux of cefadroxil at Caco-2 cell monolayers while Al^3+^ ions had no effect. The interaction of cephalosporins with the metal ions could suggest negative effects of some metal ions on the clinical aspects of small intestinal peptide and drug transport. Finally, the effect of Zn^2+^, Cu^2+^ and Al^3+^ ions on the antibacterial activity of cefadroxil was tested. It was found that there is no significant difference between the activity of cefadroxil and the cefadroxil metal ion complexes studied against the investigated sensitive bacterial species.

## 1. Introduction

Cefadroxil is a semi-synthetic first generation cephalosporin which has been introduced into clinical practice and recommended for oral use. Similar to cefalexin and cefradine, cefadroxil has been shown to have an antibacterial activity against 602 clinical isolates of different kinds of bacteria [1]. Most of the antibiotics, including cephalosporins, and their decomposition products contain electron donor groups that can bind naturally-occurring metal ions *in vivo*. Metal ions form chelates with these antibiotics and reduce their intestinal absorption. It was reported that cefadroxil can form complexes with different metal ions [1,2]. The interaction between cefadroxil and different metal ions was characterized by capillary electrophoresis [3].

In the gastrointestinal tract, dietary proteins are degraded into a mixture of free amino acids and small peptides. The H^+^-coupled peptide transporters (PEPT1 and PEPT2) expressed in the brush border membranes of the intestinal and renal epithelial cells, respectively, mediate the absorption of di- and tripeptides and play important nutritional roles for protein homeostasis [4,5]. Within the cell, most of the dipeptides and tripeptides are hydrolyzed by cytosolic peptidases and are released as free amino acids into circulation [6]. Due to their broad substrate specificity, PEPT1 and PEPT2 can accept several peptide-like drugs structurally related to small peptides such as oral β-lactam antibiotics, the anticancer agent bestatin and the antiviral drug val-acyclovir [4,7,8]. It has been well established that the good oral availability of certain β-lactams, in particular of the cephalosporins, is a consequence of their ability to use the intestinal peptide transporter for the uptake into circulation [9,10]. Because of this characteristic, cefadroxil has been used in expression cloning to isolate the cDNA clones that encode PEPT1 and PEPT2 [11].

Most importantly, Caco-2 cells constitutively express the peptide transporter PEPT1 in their apical membrane and can be used for transport measurements of β-lactam antibiotics [9]. There are several publications concerning the interaction of inorganic compounds with peptide transporters [8,12,13,14]. In the present study, we examined the effect of different metal ions on the transport of Gly-Sar and cefadroxil in Caco-2 cells as well as their effect on the antimicrobial activity of cefadroxil.

## 2. Results and Discussion

### 2.1. Uptake and transport studies

**Figure 1:**
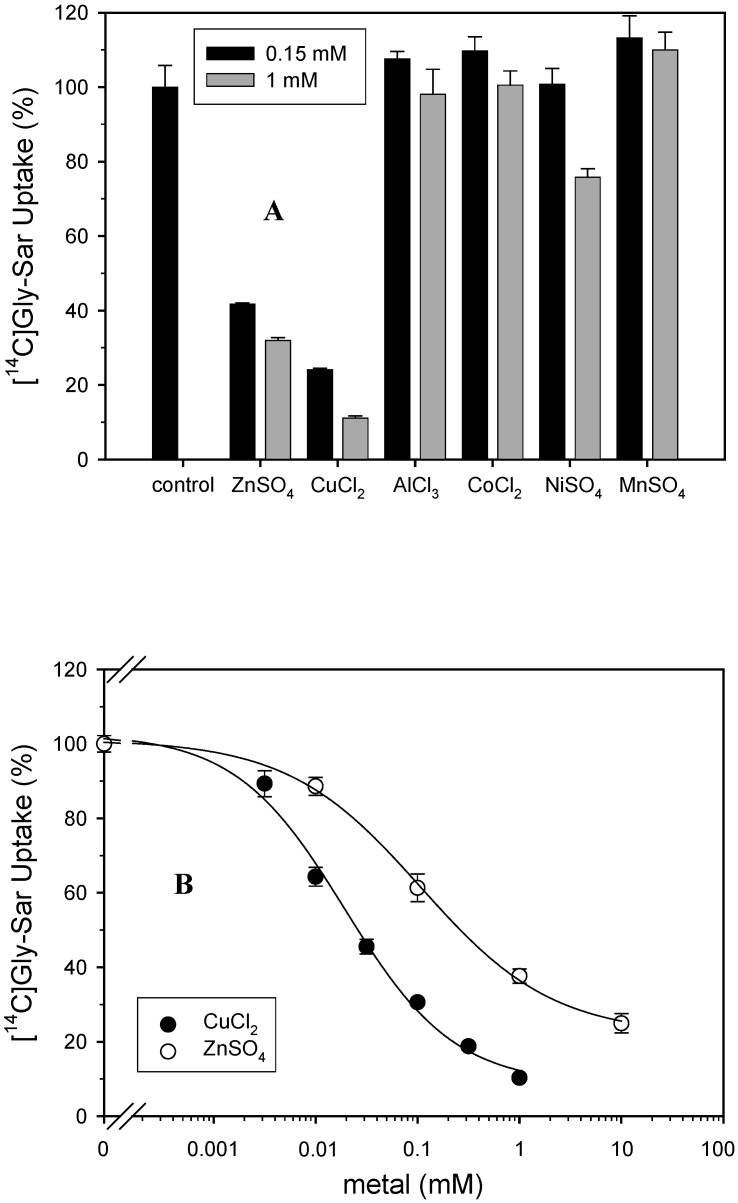
Effect of different metals salts on [^14^C]Gly-Sar uptake in Caco-2 cells. **A**: Uptake of [^14^C]Gly-Sar (10 µM) was measured in Caco-2 cells (pH 6.0, 10 min, n = 3 - 4) in the absence and presence of different metals salts (0.15 and 1 mM). Uptake of [^14^C]Gly-Sar measured in the absence of the inhibitors was taken as 100% (control). **B**: Uptake of [^14^C]Gly-Sar (10 µM) was measured in Caco-2 cells (pH 6.0, 10 min, n = 3 - 4) in the presence of increasing concentrations of ZnSO_4_ and CuCl_2_ (0–10 mM). Uptake of Gly-Sar measured in the absence of inhibitor 107 ± 23 and 19 ± 5 µM for [^14^C]Gly-Sar in presence of zinc and copper, respectively.

Different studies suggest out that substrates of PEPT1 and metal ions interact with each other during the intestinal absorption [15,16]. So, the question arises whether these metal ions affect drug absorption by chelating with drugs or by modulating the function of PEPT1. In the current study, the inhibition of six different di and tri-valent cations at two concentrations on the [^14^C]Gly-Sar uptake via PEPT1 in Caco-2 cells was determined (Figure 1A).

**Figure 2:**
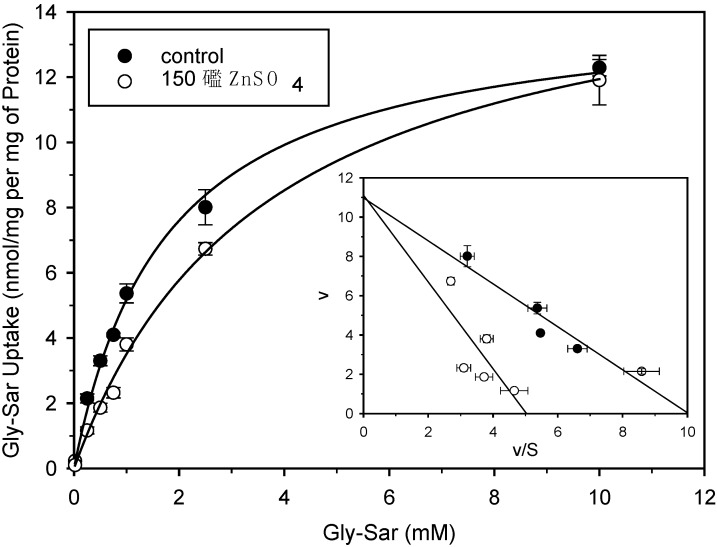
Effects of zinc sulphate on the saturation kinetics of Gly-Sar uptake into Caco-2 cells. Uptake of [^14^C]Gly-Sar was measured at pH 6.0 for 10 min. The results represent saturable uptake values after correction for the non-saturable component. Inset: Eadie-Hofstee transformations of the data(v = uptake rate in nmol 10 min^-1^ mg protein^-1^; S = Gly-Sar in mM). n = 4.

In order to determine the effects of zinc on the kinetic parameters of Gly-Sar uptake, we studied the relationship between Gly-Sar uptake rates and extracellular Gly-Sar concentrations in Caco-2 cells (Figure 2). Kinetic parameters were calculated according to the Michaelis-Menten equation. In the presence of zinc at a concentration close to its K_i_ value (150 µM) the K_t_ of Gly-Sar uptake was increased 2-fold (1.7 ± 1.1 mM to 3.6 ± 0.3 mM), whereas the V_max_ values were not affected (14.3 ± 0.4 nmol mg of protein^-1^ 10 min^-1^ versus 16.3 ± 0.6 nmol mg of protein^-1^ 10 min^-1^). These results suggest that zinc sulphate inhibited Gly-Sar uptake by PEPT1 in a competitive manner.

**Figure 3:**
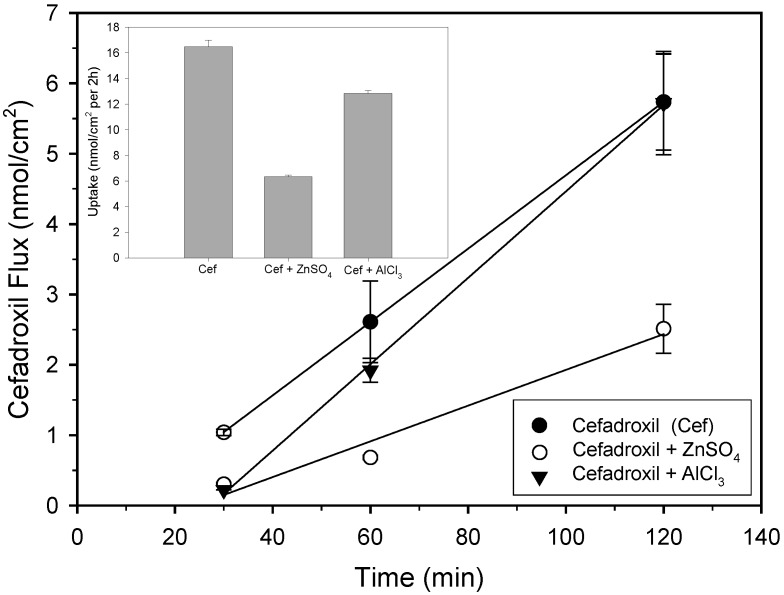
Effect of ZnSO_4_ and AlCl_3_ on total transepithelial flux and intracellular accumulation of cefadroxil at Caco-2 cell monolayers. Cefadroxil (1 mM) was added to the apical (donor) compartment of the Transwell^®^ chambers in uptake buffer (pH 6.0) with or without 1 mM ZnSO_4_ or AlCl_3_. After the time intervals indicated, samples were taken from the receiver compartment (pH 7.5) and replaced with buffer. Inset: Cefadroxil content in cells on filters cut out of the plastic inserts after the flux measurements. The amount of cefadroxil in the basolateral compartment and in the cells was measured with HPLC. Data are shown as means ± S.E., n = 3.

To investigate the effect of metal ions on the total net transport of cefadroxil, experiments using Caco-2 cells cultured on permeable filters were performed. The integrity of the monolayers was evaluated by measuring the Transepithelial Electrical Resistance (TEER). In the present study, the mean of the TEER values of the Caco-2 monolayers was 629 ± 11 Ω^.^cm^2^. Time courses of cefadroxil flux across Caco-2 cell monolayers in presence and absence of metal ions are shown in Figure 3.

The amount of the drug transported from the apical to basal side increased linearly with time. Transported amounts of cefadroxil (1 mM) per 60 min were 2.60 ± 0.15 (control), 0.91 ± 0.04 (ZnSO_4_, 1 mM) and 2.01 ± 0.13 nmol cm^-2^ h^-1^ (AlCl_3_, 1 mM), respectively. Hence, the cefadroxil flux in the presence of Zn^2+^ ions was reduced approximately 3-fold and in the presence of Al^3+^ only 1.3 times. We could not investigate the effect of Cu^2+^ ions on the flux of cefadroxil due to the instability of its solution with cefadroxil at 37 °C. In a previous study [3] we determined the association constant of the interaction of cefadroxil with zinc and with aluminium ions. Our results indicated that the reaction strength between cefadroxil and metals used follows the order: Zn^2+^ > Al^3+^ with association constants of 1546.52 and 451.57 L/mmol respectively. This corresponds well with the effect of these ions on cefadroxil flux. The effect of zinc sulfate on the transepithelial flux in Caco-2 cells was also measured for three cephalosporins with different chemical structures. The results showed that addition of zinc sulphate decreased the flux of cephalosporins with free carboxylic function at cephem-ring system, like cephalexin (1.7-fold) and cephalothin (2.7-fold). The flux of esterified cefuroximaxetil was not affected.

It has been reported that metal ions can affect the absorption of several organic solutes by modulating their respective transporters. For example, zinc exhibited inhibitory effects on the glucose transporter [17], threonine transporter [18] and Na^+^,K^+^-ATPase [15]. These reports suggest that metal ions interact with these transporters. Daniel and Adibi showed that the incubation with metal ions modulates the renal transport of dipeptides and cephalexin by PEPT1 and PEPT2 [8]. In contrast, Ueno *et al.* described inhibition of the oral absorption of cephalosporin cefdinir due to complex formation of the transporter substrate with iron(ΙΙ) ions [14].

As for the opposite effect, the absorption of metal ions may also be affected by organic solutes. For example, Steinhardt and Adibi reported that the intestinal zinc absorption was increased addition of glycylleucine addition, whereas the free amino acids had no influence [19]. Tacnet and coworkers postulated that zinc ions can be transported by a complex formation with the PEPT1 substrate Gly-Gly-His into the cells [13]. According to Glahn and Campen the intestinal absorption of iron is influenced by amino acids and peptides from the protein digestion [16].

Therefore, the following three mechanisms are conceivable to explain the effects measured in this study: First, zinc ions could bind at an allosteric center and modify the substrate binding site of PEPT1. However, in our kinetic analysis we found a competitive mode of inhibition of Gly-Sar transport. Second, zinc and copper ions themselves could be substrates for PEPT1. This is unlikely considering the well known substrate specificity of PEPT1. Third, zinc ions form a complex with substrates of PEPT1 and this complex has no or low affinity to PEPT1. The latter scenario is supported by the results by Tacnet and co-workers [13]: At low concentrations, the tripeptide Gly-Gly-His stimulated and at higher concentrations it inhibited zinc uptake. This can be explained by a crucial peptide/Zn^2+^ ratio. When the amount of peptide is higher than the peptide/ Zn^2+^ complex, which has a lower affinity to the peptide transporter, the complex is not transported any more, so the overall Zn uptake is reduced. With regard to our study, we conclude that zinc and copper ions form complexes with Gly-Sar and with cefadroxil thereby decreasing the concentration of free substrate in the extracellular medium. This decrease of free [^14^C]Gly-Sar concentration at increasing metal salt concentrations resulting in increasing complex formation can easily explain the decline in [^14^C]Gly-Sar uptake observed in this study (Figure 1). The results do not suggest a metal ion-PEPT1 interaction.

### 2.2. Antibacterial activity tests

Cefadroxil interferes with cell-wall synthesis of bacteria, leading to lysis of the infectious microorganisms. The antibacterial activities of cephalosporin metal ion complexes depends mainly on the type of cephalosporin used, the type of metal ion and the type of microorganism under investigation. The results of the paper disc diffusion method of the current study are shown in Table 1. 

**Table 1:** Antibacterial activity of cefadroxil and its complexes against different bacteria*.

Bacteria	Inhibition zone (mm)			
	Cefadroxil	Cef-Zn	Cef-Cu	Cef-Al
*E. coli*	11.5 ± 0.4	12.2 ± 0.3	9.2 ± 0.4	9.7 ± 0.3
*S. carnosus*	32.3 ± 0.8	33.2 ± 0.8	33.3 + 0.9	32.5 ± 1.4
*P. vulgaris*	0.0	0.0	0.0	0.0
*B. subtilis*	33.2 ± 0.6	33.8 ± 0.6	34.0 ± 0.6	34,0 ± 0.6
*P. putida*	0.0	0.0	0.0	0.0
*M. luteus*	31.0 ± 0.7	32.8 ± 0.3	32.7 ± 0.4	32.7 ± 0.4

*Antimicrobial activity of cefadroxil, in a concentration of 10 mg/mL, and its metal complexes (1:1 mmol) was tested using the paper disc diffusion method in solid Luria Bertani medium. Data are shown as mean ± S.E., n = 6.

Cefadroxil and its metal complexes showed higher antibacterial activities against the investigated sensitive Gram positive bacteria (*Staphylococcus carnosus*, *Bacillus subtilis and Micrococcus luteus*) than sensitive Gram negative ones (*Escherichia coli* W3110). While the compounds exhibited moderate effect on *Escherichia coli (Gram negative)*, *Proteus vulgaris (Gram negative)* and *Pseudomonas putida (Gram negative)* are resistant. More importantly, using ANOVA and T test, statistical analysis showed no significant differences between the antibacterial activities of cefadroxil alone and its metal ion complexes.

It was noticed that complexation of cephalexin with cadmium ions exhibited no effect on the antibacterial activity of cephalexin [20]. Also, the antibacterial activity of cefazolin didn’t affected by complexation with copper ions [21].

The antibacterial activities of Mn^2+^, Co^2+^, Ni^2+^, Cu^2+^ and Zn^2+^ complexes of cephradine was studied by Anacona and Acosta [22]. They observed that the antimicrobial activity of cephradine was decreased by metal complexation. In contrast, it has been reported that Co^2+^ and Ni^2+^ complexes of cefaclor [23] and Zn^2+^ and Cu^2+^ complexes of cephalexin [24] showed higher antibacterial activity. In our study we could not find a difference in the antibacterial activity of cefadroxil and their metal complexes. The relationship between chelation and antibacterial activity seems to be a function of steric, electronic and pharmacokinetic factors, along with mechanistic pathways [22]. 

## 3. Experimental

### 3.1. Materials

Cefadroxil monohydrate (99%) was obtained from MP Biomedicals Co. (Kayserberg, France). CuCl_2_·2H_2_O (98%) and ZnSO_4_·7H_2_O (99%) were purchased from Sigma-Aldrich GmbH. CoCl_2_·4H_2_O (98%) was supplied by Roanal Budapest (Hungary). NiSO_4_·6H_2_O (97%) was obtained from (Gruessing, Germany). AlCl_3_ (99%) and MnSO_4_ (99%) was purchased from Germed (Germany). The Caco-2 human colon carcinoma cell line was obtained from the German Collection of Microorganisms and Cell Cultures (Braunschweig, Germany). Culture media and supplements, fetal bovine serum, and the trypsin solution were purchased from Invitrogen (Karlsruhe, Germany), PAA (Pasching, Austria) or Biochrom (Berlin, Germany). [*Glycine*-1-^14^C]-Glycyl-Sarcosine ([^14^C]Gly-Sar, specific radioactivity 53 mCi/mmol) was custom synthesized by GE Healthcare (Buckinghamshire, UK). 

### 3.2. Caco-2 cell culture and uptake assessments

The details of preparation and characterisation of cefadroxil-metal complexes were mentioned in our previously published paper [3]. The Caco-2 human colon carcinoma cell line was routinely cultured with minimum essential medium supplemented with 10% fetal bovine serum, 1% nonessential amino acid solution, and gentamicin (45 µg/mL) [9]. For most experiments, the cells were seeded in 35 mm disposable Petri dishes (Sarstedt, Nümbrecht, Germany) at a density of 0.8 × 10^6^ cells per dish. The uptake was measured on the seventh day after seeding. Caco-2 cells were also cultured on permeable polycarbonate Transwell cell culture inserts (24.5 mm diameter, 3 µm pore size, Costar GmbH, Bodenheim, Germany). Subcultures were started at a cell density of 43,000 cells/cm^2^ and cultured for 20–25 days. Uptake of [14C]Gly-Sar in cells cultured on plastic dishes was assessed as described elsewhere [9]. Because the peptide transporter is transporting the dipeptide in symport with a H+ it needs a pH-gradient (pH 6.0 outside of the cells and around 7.3 inside the cells). The transport of the peptide is linear for at least 10 min, so we used 10 min incubation time for the experiments (the experiment for this was done several years ago by Brandsch *et al*. [25]). The uptake buffer contained 25 mM 2-(*N*-morpholino) ethanesulfonic acid/tris (hydroxymethyl) aminomethane (Mes/Tris pH 6.0 ), 140 mM NaCl, 5.4 mM KCl, 1.8 mM CaCl_2_, 0.8 mM MgSO_4_, 5 mM glucose, 10 µM [14C]Gly-Sar, and increasing concentrations of unlabeled inhibitors (Gly-Sar, cefadroxil, metal ions). After incubation for 10 min, the cells were quickly washed, solubilised in 1 mL of Igepal® Ca-630 (0.5% v/v) in buffer (50 mM Tris/HCl, pH 9.0, 140 mM NaCl, 1.5 mM MgSO_4_) and prepared for liquid scintillation spectrometry. Transepithelial flux of cefadroxil across cells cultured on permeable filters was measured as described previously [9]. Uptake/flux was started by adding buffer (pH 6.0, 1.5 mL) containing cefadroxil to the donor side. At time intervals of 10–120 min, samples were taken from the receiver compartment (pH 7.5). After 2 h, the filters with the cells were washed, cut out of the plastic insert, transferred to vials containing 0.5 mL deionized water, 0.5 mL trichloroacetic acid solution (20%) was added, centrifuged with 2,000 rpm for 10 min and measured by HPLC.

### 3.3. HPLC analysis

Cefadroxil concentration was quantified in extracellular uptake medium, samples from flux studies, and in cell contents according to the laboratory standard HPLC (HPLC-Waters Delta 600, Milfold, MA, USA) with a Waters 2996 Photodiode array detector, Waters Pump Delta 600, Waters 600 Controller, Autosampler Waters 717 plus and an Eurospher column (100-5, C 18, 250 × 4 mm. The eluent was 10% acetonitrile/ 90% citrate buffer pH 3.0 at flow rate of 1.0 mL/min and an injection volume of 10 μL. UV detection was done at 270 nm.

### 3.4. Antimicrobial activity test

Antimicrobial activity of cefadroxil and its metal complexes was determined using three strains of gram positive [*Staphylococcus carnosus* (DSM 20501), *Micrococcus luteus* (DSM 20030) and *Bacillus subtilis* (DSM 704) and three of gram negative [*Escherichia coli* W3110 (Bachmann, B.J. 1972. Pedigrees of some mutant strains of *Escherichia coli* K-12. Bacteriol. Rev. 36:525-557), *Proteus vulgaris* (DSM 13387)] and *Pseudomonas putida* (DSM 291)] bacteria (DSMZ, Braunschweig, Germany). The inhibition zones were determined using the paper disc diffusion method on solid Luria Bertani medium (Difco^TM^ Lennox; Becton Dickinson, Germany) (for *S. carnosus* and *M. luteus* 1 mM glucose was added). A pre-culture for each bacterium was incubated at 30 °C, 250 rpm, for 17 h, then diluted 1:400 in fresh medium and incubated for 2 h at 30 °C, 250 rpm. 500 µL of this 2 h-culture were plated onto LB agar, dried and paper discs with 10 µL of the complex solution were applied. The complexes were tested at a concentration of 10 mg/mL in Tris-HCl (pH 6). For control, Tris-HCl (pH 6) without drug and metal salts was applied to the paper discs. After 20 h at 30 °C the inhibition zone as diameter was measured in mm (n = 6).

### 3.5. Calculations and Statistics

All data are given as the mean ± S.E. of three to six independent experiments. The kinetic parameters were calculated by non-linear regression methods and confirmed by linear regression of the respective Eadie-Hofstee plots. IC_50_ values (*i.e.*, concentration of inhibitors necessary to inhibit 50% of carrier-mediated [^14^C]Gly-Sar uptake) were determined by non-linear regression using the logistical equation for an asymmetric sigmoid (allosteric Hill kinetics): y = Min + (Max-Min)/(1 + (X/IC_50_)^-P^) where Max is the initial Y value, Min the final Y value and the power P represents Hills’ coefficient. Significant differences between the antibacterial activities of cefadroxil and its metal ion complexes were statistically analyzed by using ANOVA and T test.

## 4. Conclusion

Zn^2+^ ions inhibit the intestinal transport of cefadroxil by forming strong complexes with cefadroxil. Al^3+^ ions showed no inhibitory effect on the intestinal transport of cefadroxil across Caco-2 cell which may be attributed to formation of weaker complex with cefadroxil. These complexes have low or no affinity to PEPT1. Although complexation with metal ions inhibits the transport of cefadroxil, it has no effect on its antibacterial activities. The present observations could suggest negative effects of some metal ions on the nutritional and clinical aspects of small intestinal peptide and drug transport. For example, if zinc ions or copper ions co-administered with peptide-like drugs such as oral β-lactam antibiotics. The oral bioavailability of peptide-like drugs may be impaired.
